# Effect of root conditioning agents on teeth periodontal using Scanning electron microscopy

**DOI:** 10.6026/973206300211683

**Published:** 2025-06-30

**Authors:** Balaji V.R., Manikandan D., Varshiny M.D.

**Affiliations:** 1 Department of Periodontics & Implant Dentistry, CSI College of Dental Sciences and Research, Madurai, Recognised by Government of India and Dental Council Of India, New Delhi, Affliated to The Tamil Nadu Dr MGR Medical University, Chennai, India

**Keywords:** Root Conditioning, periodontal disease, Scanning Electron Microscopy (SEM), EDTA, citric acid, Tetracycline HCL, Phosphoric acid, Hyaluronic acid

## Abstract

Mechanical instrumentation of root surfaces results in the formation of a smear layer that may impede the reattachment of connective tissue. Root conditioning agents are employed to remove the smear layer, expose collagen fibres and facilitate improved tissue regeneration. Therefore, it is of interest to assess the efficacy of five root conditioning agents-20% citric acid, 17% EDTA, 37% phosphoric acid, 250 mg/mL tetracycline hydrochloride and 0.8% hyaluronic acid-using scanning electron microscopy. Tetracycline hydrochloride showed the greatest efficacy, exhibiting a significantly higher number of patent dentinal tubules compared to other agents. Thus, root surface conditioning enhances biocompatibility, promotes fibroblast adhesion and contributes to favourable periodontal healing and successful surgical outcomes.

## Background:

Periodontal diseases often lead to the exposure of root surfaces, resulting in bacterial colonization and structural alterations that hinder the healing process. The primary goal of periodontal therapy is to restore the lost periodontium and transform periodontally affected root surfaces into a biologically favorable substrate that supports epithelial and connective tissue cell adhesion and attachment [1]. Scaling and root planing (SRP) is considered the gold standard for the non-surgical treatment of chronic periodontitis. However, its ability to completely remove the smear layer from the root surface is limited [2]. Mechanical decontamination alone is insufficient, as bacterial toxins may persist within the root surface and the instrumented area inevitably becomes covered by a smear layer. This layer composed of dental calculus remnants, contaminated cementum and sub gingival plaque, acts as a physical barrier, preventing direct interaction between the periodontal tissues and root surface, thereby inhibiting new attachment formation [3, 4-5]. The presence of a residual smear layer can significantly interfere with the healing process by obstructing the reattachment of periodontal cells to the root surface, which is essential for regeneration. To enhance the effectiveness of SRP and promote optimal healing, adjunctive therapeutic approaches may be required.

Various root conditioning agents, including ethylenediaminetetraacetic acid (EDTA), citric acid, tetracycline hydrochloride and hyaluronic acid, are commonly used in chemical treatments to remove the smear layer and expose collagen fibers on the root surface [6, 7, 8- 9]. These agents help expose dentin collagen and cementum-bound proteins, facilitating the elimination of retained bacterial toxins from the altered root surfaces. Additionally, they enlarge dentinal tubules, creating an environment that supports connective tissue healing and attachment [10]. As a result, root conditioning is recommended as an adjunct to mechanical root surface debridement to improve periodontal regeneration by enhancing fibroblast adhesion and promoting tissue repair. Therefore, it is of interest to compare the efficacy of different root conditioning agents on periodontally involved human teeth using scanning electron microscopy (SEM).

## Materials and Methods:

## Sample collection:

Six extracted human teeth with periodontally affected root surfaces were selected. Six teeth was sectioned into two halves and made into thin sections. The study group comprised of 12 dentin samples, with two samples in each group.

## Test GROUP I:

After scaling and root planing with curettes, specimens were treated with root conditioning agents and stored in distilled water.

In Group Ia - Specimens were treated with 20% Citric acid

In Group Ib - Specimens were treated with 17% EDTA

In Group Ic - Specimens were treated with 37% Phosphoric acid

In Group Id - Specimens were treated with 0.8% Hyaluronic acid

In Group Ie - Specimens were treated with 250mg/mL Tetracycline HCL

The solutions of 20% citric acid, 17% ethylene diamine tetra acetic acid (EDTA), 37% Phosphoric acid, 0.8% Hyaluronic acid, 250mg/mL of Tetracycline HCL were applied to the root surfaces with applicator tips for 5 minutes using "Active Burnishing Technique" (Figure 1 - see PDF).

## Control GROUP II:

Scaling and root planing were the only procedures performed in control group and the specimens were stored in distilled water.

## Scanning electron microscopy (SEM) analysis:

After treatment, the samples were sent to laboratory for SEM analysis under magnifications of 1000x, 3000x, 5000x (Figure 2-7 - see PDF).

## Statistical analysis:

One- Way analysis of variance (ANOVA) was used for intergroup and intragroup comparison. Tukeys post-hoc test was used for multiple pairwise comparisons. The statistical analysis was carried out using IBM SPSS version 20.0 (IBM Corp. Released 2011. IBM SPSS Statistics for Windows, Version 20.0. Armonk, NY: IBM Corp).

## Results:

The patency of the dentinal tubules was visually assessed at 5000x magnification (Table 1 - see PDF). Among the tested agents, 250 mg/mL Tetracycline demonstrated the highest mean value (42.00 ± 4.24), indicating superior root conditioning efficacy.

37% Phosphoric Acid (H_3_PO_4_) followed with a mean value of 26.00 ± 5.65. 17% EDTA (Ethylene Diamine Tetraacetic Acid) and 0.8% Hyaluronic Acid exhibited moderate efficacy with mean values of 19.50 ± 2.12 and 17.50 ± 3.53, respectively.20% Citric Acid showed a lower mean value (15.00 ± 1.41), whereas non-scaled dentin surfaces had the lowest mean value (4.00 ± 2.82), highlighting the significance of root conditioning in periodontal treatment. The confidence interval (95% CI) ranged widely for some agents, with Tetracycline showing a relatively broad range (3.88 to 80.11), reflecting variations in its efficacy. Similarly, Phosphoric Acid and Hyaluronic Acid had broad confidence intervals, indicating variability in their effects on dentin surfaces. In contrast, Citric Acid and EDTA demonstrated more consistent outcomes with narrower confidence intervals. These findings suggest that 250 mg/mL Tetracycline HCL may be the most effective agent for root conditioning, while other agents show varying degrees of effectiveness (Table 2 - see PDF). The results of one-way analysis of variance (ANOVA) demonstrated a statistically significant difference among the groups (F = 25.086, p = 0.001), indicating that the effectiveness of root conditioning agents varied considerably. The between-group variance (Sum of Squares = 1609.667, df = 5, Mean Square = 321.933) was much higher than the within-group variance (Sum of Squares = 77.000, df = 6, Mean Square = 12.833), suggesting that the differences observed across groups were due to the specific effects of the conditioning agents rather than random variation. Among the tested agents, 250 mg Tetracycline exhibited the highest number of patent dentinal tubules, indicating its superior efficacy in modifying the dentin surface. In contrast, the control group (non-scaled dentin surfaces) showed the highest number of occluded dentinal tubules, reinforcing the importance of root conditioning in periodontal therapy. The significant p-value (p = 0.001) confirms that the observed differences were statistically meaningful (Table 3 - see PDF) (Table 4 - see PDF).

## Discussion:

The present study aimed to compare the efficacy of various root conditioning agents in widening the dentinal tubules of periodontally involved human teeth using Scanning Electron Microscopy (SEM). Root surface bio modification was first proposed about 50 years ago by Register and Burdick [11]. It aims to counteract the harmful effects of plaque, calculus and contaminated cementum on the root surface to facilitate regenerative therapies. This process can be achieved through mechanical, chemical, or combined approaches. Mechanical instrumentation smooth out irregularities reduces root convexity and minimizes cementum toxicity. In contrast, chemical treatment aims to restore a biologically compatible root surface by counteracting the structural and biochemical damage resulting from exposure to the oral environment and bacterial endotoxins. These detrimental changes may include compromised collagen fiber insertion, alterations in mineral density and surface composition, bacterial contamination and endotoxin presence [12, 13- 14].

Given that the root surface serves as a wound margin during regeneration, conditioning it with chemical modifying agents may enhance cell attachment and fiber integration, ultimately promoting periodontal healing. The findings revealed significant differences among the tested agents, as demonstrated by the ANOVA results (F = 25.086, p = 0.001). The ability of a conditioning agent to modify the root surface plays a crucial role in periodontal regeneration by enhancing fibrin adhesion, improving epithelial attachment and reducing bacterial penetration. Among the tested agents, 250 mg/mL Tetracycline HCL solution exhibited the highest number of patent dentinal tubules, suggesting its superior root conditioning effect. This aligns with previous studies that have shown Tetracycline to effectively remove the smear layer, expose collagen fibers and promote fibroblast attachment. The acidic nature of Tetracycline helps in demineralizing the dentin surface, thereby increasing the permeability of the root surface and enhancing adhesion [15].

37% Phosphoric Acid (H_3_PO_4_) also demonstrated a considerable effect on widening the dentinal tubule, though it was less effective than Tetracycline. Its ability to dissolve the smear layer and open dentinal tubules is well-documented, but the extent of its demineralization may sometimes lead to excessive erosion, which can negatively impact attachment. 17% EDTA (Ethylene Diamine Tetra acetic Acid) and 0.8% Hyaluronic Acid showed moderate efficacy. EDTA is a known chelating agent that selectively removes the smear layer without excessive demineralization, making it beneficial for periodontal therapy. Hyaluronic Acid, a biocompatible agent with anti-inflammatory and wound-healing properties, demonstrated promising results, suggesting its potential use as an adjunctive conditioning agent. 20% Citric Acid had a lower efficacy compared to the aforementioned agents. While Citric Acid is traditionally used to condition root surfaces by exposing collagen fibers, its effectiveness in this study was relatively limited. This could be attributed to variations in pH, or dentinal surface composition [16]. The control group (non-scaled dentin surfaces) exhibited the highest number of sealed dentinal tubules, reinforcing the importance of root conditioning in periodontal treatment. Unconditioned dentin surfaces often retain smear layers that hinder cellular attachment and regenerative processes, emphasizing the necessity of using decontaminating agents to enhance periodontal healing. The wide range of confidence intervals observed for some agents suggests variability in their effects, which could be attributed to differences in pH and dentin composition. Future studies with larger sample sizes could provide more definitive conclusions regarding the optimal root conditioning agent.

## Conclusion:

Thus, study concluded that all the agents were effective in removing the smear layer however the number of patent and wider diameter dentinal tubules in tetracycline was higher than the other agents.

## Clinical significance:

Root conditioning enhances biocompatibility by removing the smear layer, exposing collagen fibers and reducing microbial contamination, promoting fibroblast attachment and periodontal healing. It facilitates new attachment formation by optimizing root surface characteristics, supporting PDL cell migration and differentiation. Additionally, it aids in periodontal surgery success by improving tissue adaptation, reducing sensitivity and fostering optimal healing responses.

## Abbreviations:

EDTA - Ethylenediaminetetraacetic acid

SEM - Scanning Electron Microscopy

SRP - Scaling and Root Planing

ANOVA - Analysis of Variance

CI - Confidence Interval
